# Special Issue on Testis Cancer

**DOI:** 10.1007/s00345-022-03957-w

**Published:** 2022-02-28

**Authors:** Daniel Nettersheim, Peter Albers

**Affiliations:** 1 Department of Urology, Urological Research Laboratory, Translational UroOncology, Medical Faculty and University Hospital Düsseldorf, Heinrich Heine University Düsseldorf, Düsseldorf, Germany; 2 Department of Urology, Düsseldorf University Hospital, Medical Faculty, Düsseldorf, Germany

Germ cell tumors of the testis are rare cancers with—for unknown reasons—rising incidence in industrialized countries affecting young men in their reproductive ages peaking at 20–30 and 30–40 years depending on histology. These special circumstances demand different diagnostic and monitoring tools, different counseling and different multidisciplinary treatment goals as compared to classical oncology at elderly ages.

First, nearly all patients can be cured, some of them by orchidectomy alone, some by a combination of cisplatin-based therapy and surgery. This demands precautions in the treatment recommendations focusing on long-term rather than short-term toxicity in this young patients’ population and alternatives to the cisplatin-based therapy are warranted. Second, if spreading at all, germ cell tumors remain largely in the lymphatic drainage system and hematogenous spread is late, comparatively rare, and dependent on presence of special histologic components, like choriocarcinoma or yolk-sac tumor. This asks for very thoughtful and precise diagnosis and staging before recommending treatment. Third, a small entity of germ cell tumors harbors treatment-resistant cell clones, which need to be identified as early as possible and characterized on a molecular level to identify the underlying mechanisms of therapy-resistance. Even patients with such resistant disease patterns can be cured. Lastly, all this implies that physicians treating patients with germ cell tumors need to be counseled, second opinion before treatment should be standard and advanced metastatic patients need to be treated in highly specialized centers.

In this special issue, many of the above-mentioned prerequisites to treat patients suffering from germ cell tumors with cutting-edge knowledge and technology are presented by international experts in the field. The modern diagnostic work-up as presented by Zengerling [1], Belge [2], and Brandt [3] et al. will soon include not only magnetic resonance imaging to avoid diagnostic toxicity in young ages, but will also include new tumor markers, like microRNA371a-3p, for better non-invasive staging. The consequence of this more precise staging possibilities may be the broader indication for local and less traumatic treatment options like robotic RPLND and resection of small seminoma metastases as presented by Hamilton [4] and Daneshmand et al.[5]. Additional preoperative imaging calculations (“radiomics”) may aid in surgical planning as outlined by Nini et al.[6]. Furthermore, the fruitful collaboration of dedicated medical oncologists and specialized surgeons may improve the outcome of patients with resistant disease as presented by Oing [7], and Hiester et al. [8]. Lastly, Bremmer and Nettersheim et al. [9] identified putative factors of cisplatin resistance on proteome level by mass spectrometry. Taken together, this special issue presents up-to-date knowledge and future perspectives on the management and research of patients with germ cell tumors. These developments will influence oncology. As such, testis cancer remains a role model of oncology.
